# Hydrazone fluorescent sensors for the monitoring of toxic metals involved in human health from 2014–2024

**DOI:** 10.1039/d4ra09068c

**Published:** 2025-02-04

**Authors:** Alexander Ciupa

**Affiliations:** a Materials Innovation Factory, University of Liverpool 51 Oxford Street Liverpool L7 3NY UK ciupa@liverpool.ac.uk

## Abstract

Hydrazone-based fluorescent sensors have been instrumental for the detection of toxic metals over the past decade due to their ease of synthesis and unique properties. This review summaries the diverse range of sensors reported for toxic metals (Al^3+^, Fe^3+^, Cu^2+^, Zn^2+^ and Hg^2+^) highlighting the key role this class of sensors will play in the foreseeable future.

## Abstract 2

Hydrazone-based fluorescent sensors have been instrumental for the detection of toxic metals over the past decade due to their ease of synthesis and unique properties.

## Graphical abstract

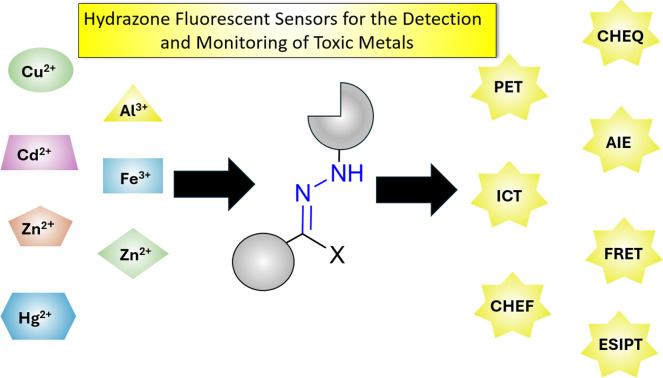

## 1 Introduction

**Scheme 1:**
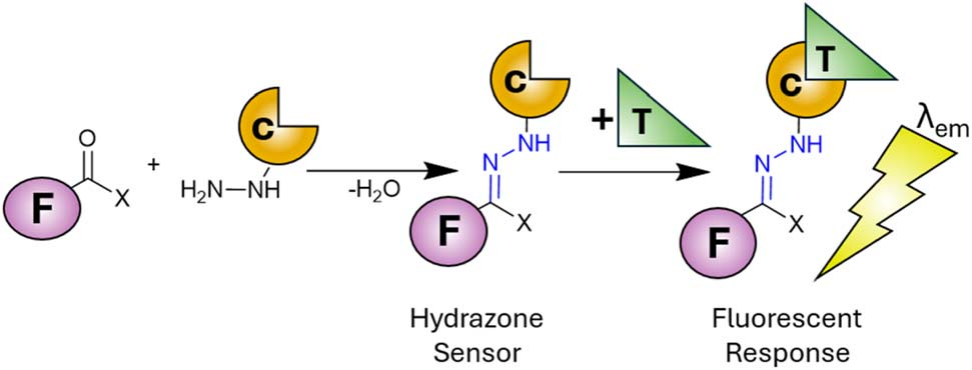
Condensation reaction between carbonyl and hydrazine to produce a hydrazone (in blue). F: fluorophore, C: chelation unit, T: target analyte and *λ*_em_ is fluorescence emission.

There are twenty essential metals for the maintenance of human life^1^ ranging from group one alkali metals (Na and K), through group two alkaline metals (Mg and Ca) to the transition metals (Cr, Mn, Fe, Co, Cu, Zn and Mo). While the regular ingestion and homeostasis of these metals is critical to life, overconsumption and dysregulation leads to disease^2^ confirming the Latin phase “dosis facit venenum” (the dose makes the poison). Alongside the essential metals to life, there are several heavy metals (Cd, Hg and Pb) with well-established toxicities^3^ therefore constant surveillance in the environment and the food supply is of paramount importance. Several analytical techniques are available to meet this challenge^4^ however they often require extensive time-consuming sample preparation coupled with expensive equipment to reach the required limits of detection (LoD). Fluorescence spectroscopy offers several advantages^5,6^ over traditional techniques including rapid analysis (seconds), low limits of detection (nanomolar range) and minimal sample volume and preparation. The development of highly selective fluorescent sensors to monitor metals within *in vitro* cell cultures^7,8^ and *in vivo*^9^ is unique to fluorescence spectroscopy. This has enabled unparalleled discovery into the role of toxic metals in human health^10,11^ and will likely continue for the foreseeable future. Fluorescent sensors are typically divided into two types, a “turn on” sensor^12,13^ in which the presence of the target analyte increases fluorescence emission at a specific wavelength (*λ*_em_) or “turn off” when analyte decreases *λ*_em_.^14^ Multiple chemical scaffolds have been utilised as fluorescent sensors^15,16^ with hydrazone sensors^17,18^ widely adopted for a myriad of different metals both in the environment and in living systems. Hydrazones can easily be synthesised by a condensation reaction between a carbonyl (typically a ketone or aldehyde) with a hydrazine derivative (Scheme 1). This well-established chemistry enables a variety of fluorophores and chelation unit (F and C in Scheme 1) combinations to achieve the desired photophysical properties. Upon binding of the target analyte (T in Scheme 1) a fluorescent response (*λ*_em_) is triggered allowing detection and quantification of the analyte in question. Careful selection of the fluorophore and chelation site enables highly specific sensors functional in complex environments to be developed. The hydrazone unit is not limited to fluorescent sensors, hydrazone based molecular switches and devices,^19,20^ promising hydrazone drug candidates^21^ and its widespread use in bioconjugation^22^ highlight the importance of this versatile functional group.

This review will provide an overview of the commonly used fluorescence sensing mechanisms for hydrazone sensors while summarising hydrazone-based sensors for toxic metals over the past decade. The recent development of multi-analyte hydrazone sensors and the challenges ahead will also be discussed.

## 2 Sensing mechanisms

**Fig. 1:**
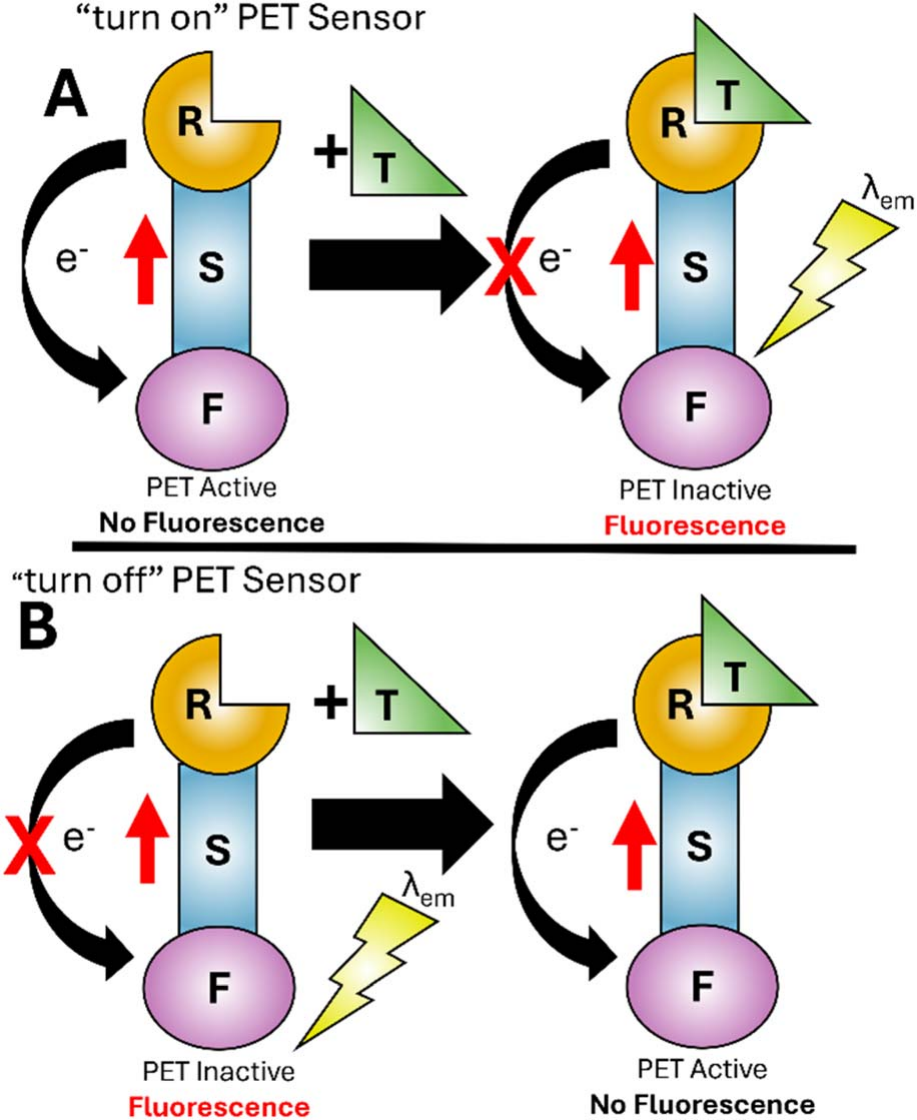
Simplified representation of the PET mechanism^23–25^ for “turn on” (panel A) and “turn off” (panel B) sensors. F: fluorophore, S: spacer, R: receptor unit, T: target analyte, red arrow indicates excitation, black arrow indicates PET.

First described by de Silva in 1985,^23,24^ a photoelectron transfer (PET) sensor consists of three components: a fluorophore (F), a spacer (S) and an receptor (R) unit (Fig. 1).^25^ The sensor undergoes excitation (red arrow in Fig. 1A) resulting in transfer of an electron from the receptor to fluorophore (black arrow in Fig. 1A) in an efficient non-radiative pathway. This is photoelectron transfer (PET) from receptor to fluorophore quenching fluorescence. On binding the target analyte (T), a conformation change alters the properties between fluorophore and receptor making PET unfavourable (red cross in Fig. 1A). Return to the ground state with the emission of *λ*_em_ is now favourable producing a “turn on” response. A “turn off” response wherein the sensor undergoes emission of *λ*_em_ in the absence of analyte can also be developed (Fig. 1B). Target analyte binding improves the properties between fluorophore and receptor activating the non-radiative PET pathway, reducing *λ*_em_ and triggering a “turn off” response. Tsien pioneered “turn on” PET sensors for intracellular Ca^2+^ monitoring.^26^

**Fig. 2:**
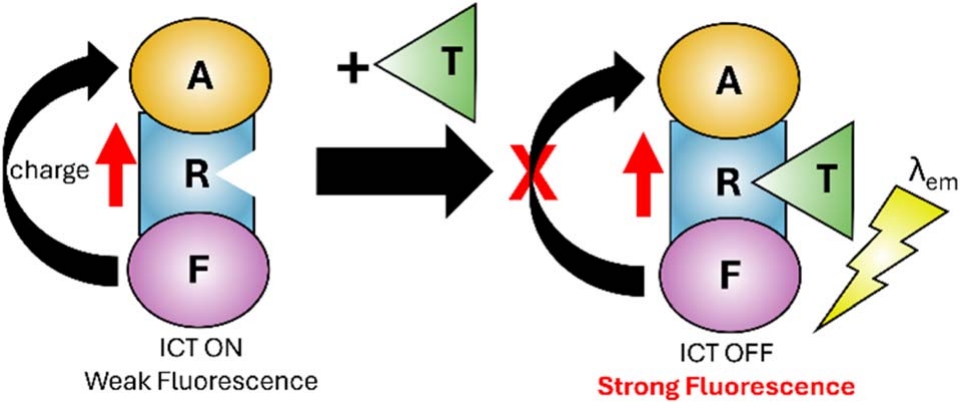
Simplified representation of the ICT mechanism.^27–29^ F: fluorophore, R: receptor, A: acceptor, T: target analyte.

An alternative to PET is internal charge transfer (ICT)^27–29^ involving an integrated fluorophore, acceptor and receptor site (F, A and R in Fig. 2). Note the receptor unit can be located on fluorophore, acceptor or a standalone unit. On excitation (red arrow in Fig. 2) the sensor can transfer charge (an electron) from electron-rich fluorophore to electron-deficient acceptor. Analyte binding changes the properties between donor and acceptor disrupting ICT. The release of *λ*_em_ is now favourable allowing return to the ground state. ICT is used for Cu^2+^ and Al^3+^ sensing.^30,31^

**Fig. 3:**
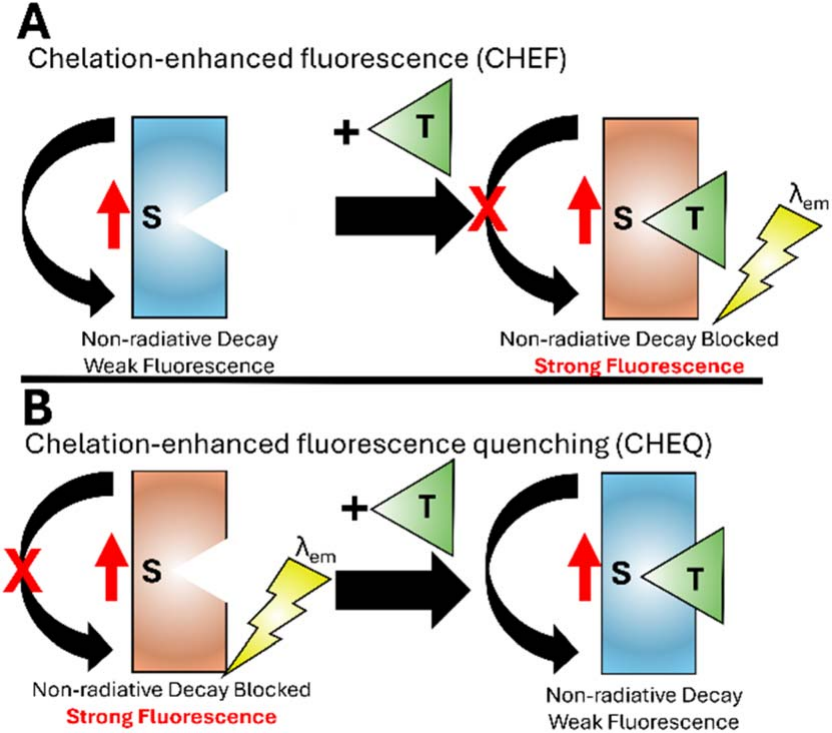
Simplified representation of the CHEF^32^ (panel A) and CHEQ^33^ mechanisms (panel B).

Chelation-enhanced fluorescence (CHEF)^32^ is a third pathway for fluorescent sensing. On excitation (red arrow Fig. 3), a sensor (S) undergoes non-radiative decay back to the ground state, typically through vibrational rotation or solvent interactions (Fig. 3). On binding the target analyte (T) a conformation change prevents this relaxation process. The only pathway available is increased *λ*_em_ often of several orders of magnitude in intensity. This mechanism has been widely employed for the development of Zn^2+^ and Al^3+^ sensors. Chelation-enhanced fluorescence quenching (CHEQ) is when the sensor itself emits fluorescence. Binding of analyte disrupts this pathway producing a “turn off” response (Fig. 3). CHEQ has been developed for Hg^2+^ sensors.^33^

**Fig. 4:**
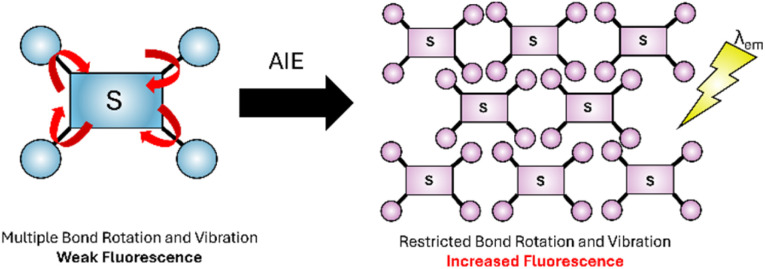
Simplified representation AIE,^34^ the dilute form (blue) with high degree of freedom (red arrows), the aggregated form has reduced bond rotation (purple).

Aggregation-induced emission (AIE), first reported by Tang^34^ can be employed for fluorescence sensing. In the dilute form the sensor has a multitude of different bond rotations and vibrations (red arrows in Fig. 4) to relax to the ground state. When aggregation is triggered, for example by analyte binding, these rotations are restricted preventing non-radiative return to the ground state and triggering the radiative release of *λ*_em_. AIE is often utilised in Fe^3+^, Cu^2+^, Zn^2+^and Hg^2+^ sensors.^35^

**Fig. 5:**
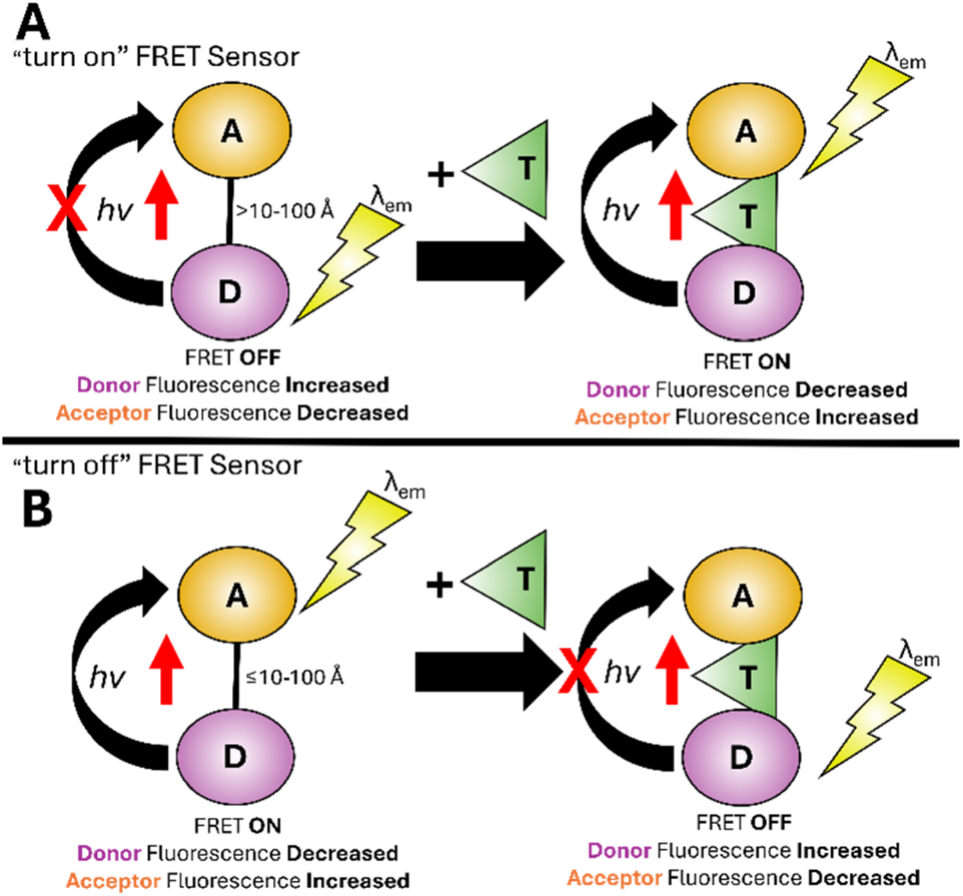
Simplified representation of the FRET mechanism.^36^ D: donor, A: acceptor, T: target analyte, *hν*: energy.

Förster Resonance Energy Transfer (FRET) first described by Förster^36^ involves two fluorophores, one acting as a donor and the other as an acceptor (D and A in Fig. 5). FRET operates over short distances, typically between 10 and 100 Å, and involves the non-radiative transfer of energy (*hν*) from the donor to acceptor if donor *λ*_em_ overlaps with acceptor *λ*_ex_. In a “turn on” FRET sensor, the donor and acceptor are unable to interact therefore we see only donor *λ*_em_ (Fig. 5A). Upon target analyte binding, a conformational change brings donor and acceptor within range to activate FRET increasing acceptor *λ*_em_ at the expense of donor *λ*_em_. In a “turn off” sensor FRET is active until analyte binding makes FRET unfavourable, for example increasing the distance between and/or preventing the overlapping of *λ*_em_ and *λ*_ex_ bands of donor and acceptor (Fig. 5B). FRET is sensitive to surroundings and has found widespread use in biomonitoring, for example proteins and peptides.^37^

**Fig. 6:**
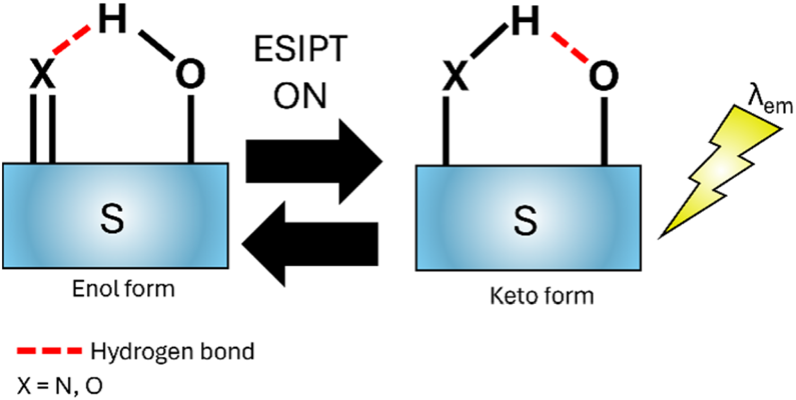
Simplified representation of the ESIPT mechanism.^38^

Excited-State Intramolecular Proton Transfer (ESIPT)^38^ was described by Weller^39^ and is applicable to sensors with intramolecular hydrogen bonding, typically a OH or NH_2_ group. Upon excitation the hydrogen donor unit (OH in Fig. 6) becomes more acidic and the hydrogen acceptor (X in Fig. 6) more basic facilitating rapid tautomerization between enol and keto states. This process involves radiative decay back to the ground state and then regeneration of the sensor to the original form. ESIPT have been widely used for biomarker detection.^40^

## 3 Sensors for Al^3+^

**Fig. 7:**
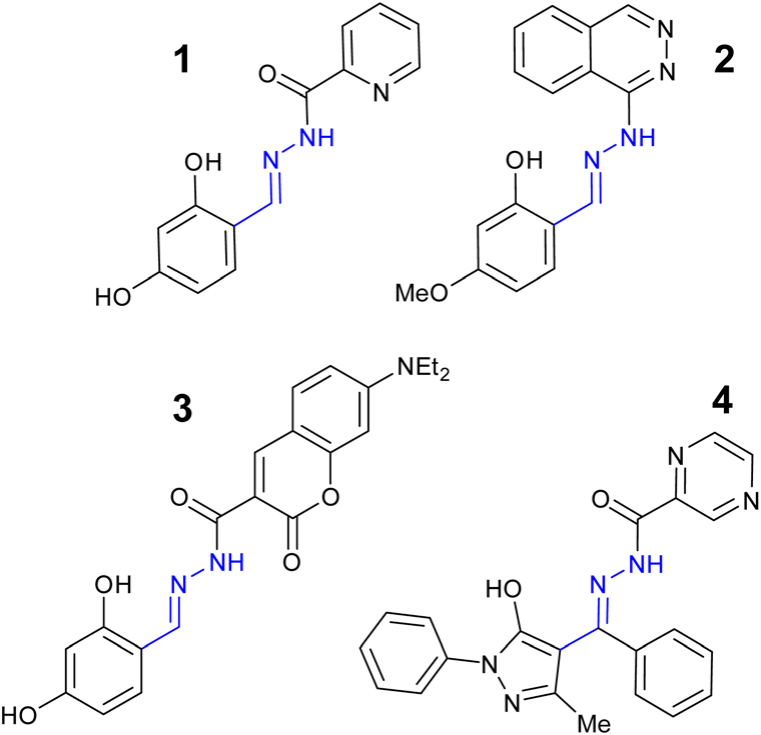
The structures of “turn on” Al^3+^ sensors 1–4.^46–49^

Aluminium is the most abundant metal in the Earth's crust and exists naturally in the trivalent form (Al^3+^) but does not have a physiological role in the maintenance of human health.^41^ Long term exposure to Al^3+^ has been linked to oxidative stress related disorders^42^ and neurodegenerative conditions such as Alzheimer's disease.^43^ The European Union (EU) aluminium drinking water limit is 7.4 μM^44^ with the World Health Organization (WHO) limit set at 33.3 μM.^45^ Acylhydrazone 1 (Fig. 7) demonstrated a “turn on” response at *λ*_em_ 456 nm with 1 : 1 binding ratio to Al^3+^*via* a ESIPT and PET mechanism.^46^ A detection limit of 21.4 nM in 9 : 1 H_2_O : DMSO was reported.^46^

**Fig. 8:**
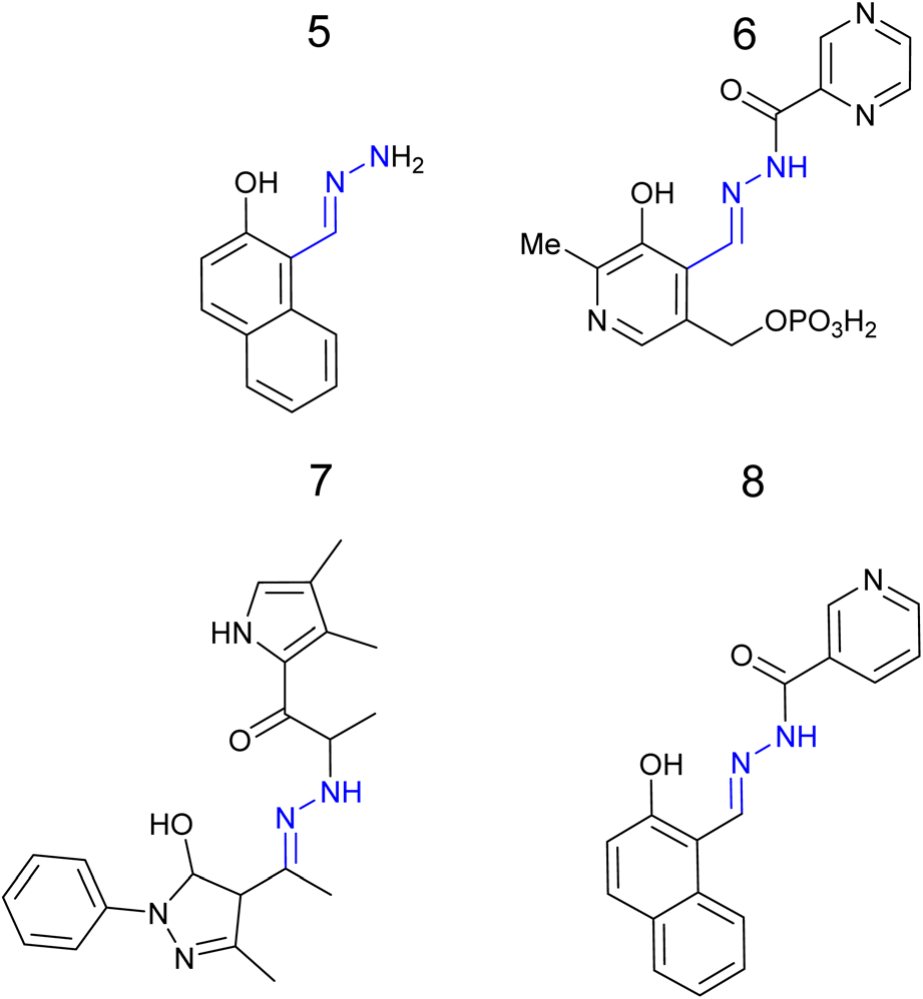
The structures of “turn on” Al^3+^ sensors 5–8.^50–53^

Quinoxaline based sensor 2 (Fig. 7) reported a similar “turn on” response for Al^3+^*via* CHEF at *λ*_em_ 460 nm with detection limit of 22 nM.^47^ Sensor 2 displayed low toxicity to normal human hepatocytes suggesting it could be a useful sensor for the monitoring of Al^3+^ in biological systems such as cell culture.^47^ Coumarin–hydrazone 3 (Fig. 7) displayed approx. 15-fold increase in *λ*_em_ at 524 nm due to CHEF with 1 : 1 binding of Al^3+^ and a LoD of 50 nM in 3 : 7 H_2_O : DMSO.^48^ Potential applications as logic gate were investigated.^48^ Pyrazine–hydrazone 4 (Fig. 7) displayed a “turn on” response at *λ*_em_ 500 nm with 1 : 1 Al^3+^ complex and a LoD 0.18 μM in 2 : 8 H_2_O : DMSO.^49^ Cell culture studies confirmed 4 could detect Al^3+^*in vitro*.^49^ Naphthalene–hydrazone 5 (Fig. 8) displayed “turn on” fluorescence enhancement at 435 nm due to AIE and ESIPT, a 1 : 1 Al^3+^ ratio and LoD of 20 nM was observed.^50^ The ability to detect Al^3+^ in both river and tap water was confirmed highlighting real-world potential of simple hydrazone sensors.^50^ Pyrazine based 6 (Fig. 8), derived from vitamin B_6_, operates almost exclusively in pure water, 99 : 1 H_2_O : DMSO, with a “turn on” signal at 456 nM due to AIE and LoD as low as 8 nM.^51^ Hydroxypyrazole 7 was developed as a “turn on” sensors for Al^3+^ at *λ*_em_ 428 nm *via* a decrease in PET and increased CHEF effect.^52^ An Al^3+^ LoD of 5 nM in ethanol and a 1 : 1 binding ratio was reported.^52^ Naphthol derived sensor 8 is a “turn on” sensor for Al^3+^ due to inhibition of ESIPT and PET forming a 1 : 1 complex with Al^3+^ and *λ*_em_ 474 nm, Al^3+^ LoD was 4 μM.^53^ In summary, multiple “turn on” sensors for Al^3+^ exploiting a variety of fluorescence mechanisms including PET, ESIPT, CHEF and AIE with LoD well below drinking water limits have been reported.

## 4 Sensors for Fe^3+^

**Fig. 9:**
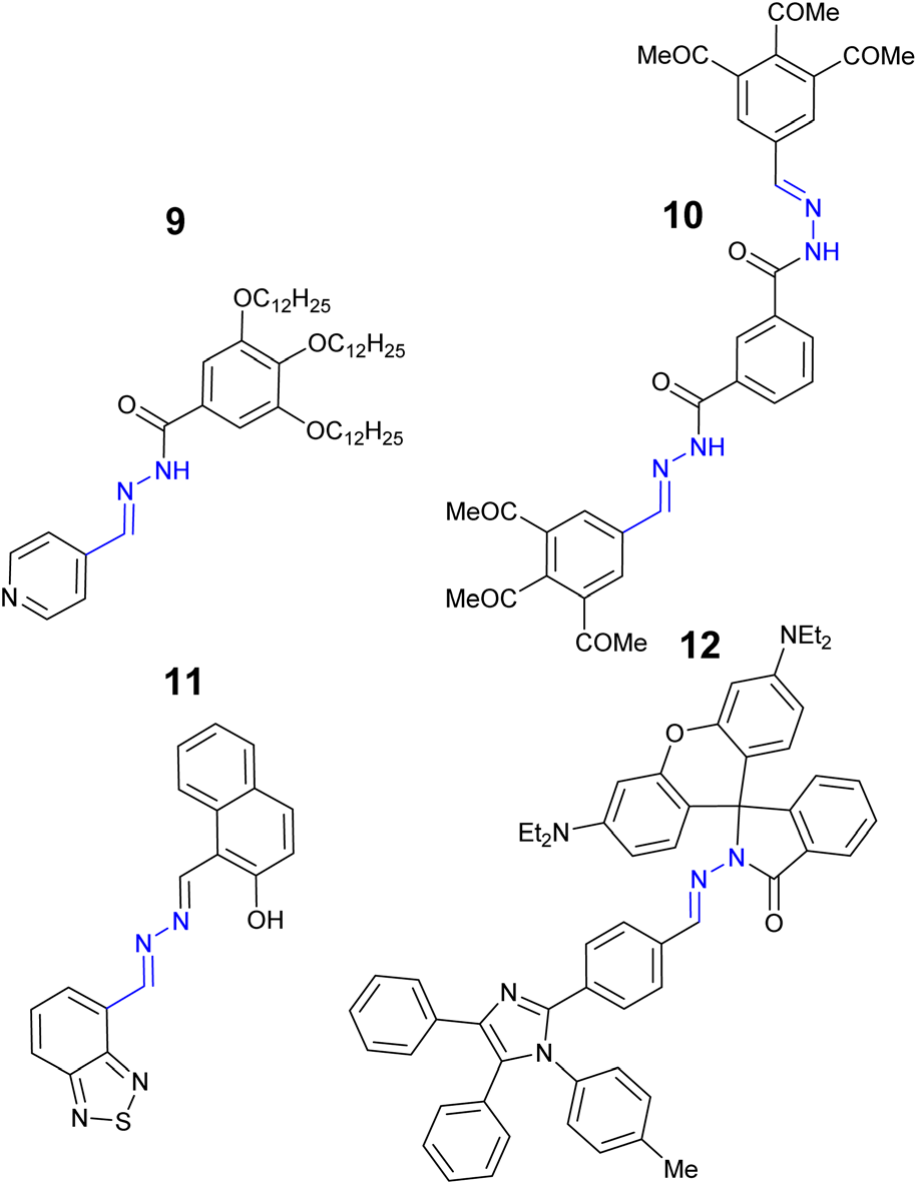
The structures of “turn on” Fe^3+^ sensors 9–12.^63–66^

Iron is the most abundant transition metal in the human body^54^ instrumental to many vital functions including the catalytic activity of enzymes,^55^ DNA synthesis and oxygen transport *via* haemoglobin.^56^ Two forms of iron predominant in life, ferric (Fe^3+^) and ferrous (Fe^2+^) iron and its close regulation is vital to health. Excess and unregulated iron is linked to oxidative stress through the Fenton reaction^57^ and medical problems including hemochromatosis and neurological diseases such as Parkinson's and Alzheimer's disease.^58,59^ The EU iron drinking water limit is 3.5 μM^60^ with the Environmental Protection Agency (EPA) in the USA limit of 5.4 μM.^61^ The development of probes to selectively detect and monitor iron both in the environment and *in vitro* is an active research area.^62^ Acylhydrazone–hydrazone 9 (Fig. 9) displayed a “turn off” response with approx. 11-fold reduction at *λ*_em_ 470 nm with Fe^3+^ due to inhibition of AIE.^63^ A LoD 1.6 μM in 1 : 4 H_2_O : THF was calculated. Sensor 10 is a “turn off” probe for Fe^3+^ also due to inhibition of AIE with a LoD of 42 nM.^64^ Naphthol–hydrazone 11 displayed a “turn off” response due to CHEQ with 1 : 1 Fe^3+^ with LoD 36 nM.^65^ The ability to monitor and track Fe^3+^ in a human prostate cell lines was reported.^65^ Rhodamine–hydrazone 12 displayed a “turn on” response to Fe^3+^ at *λ*_em_ 579 nm with Fe^3+^ and LoD as low at 11 nM in a 7 : 3 MeCN : H_2_O solution.^66^

## 5 Sensors for Cu^2+^

**Fig. 10:**
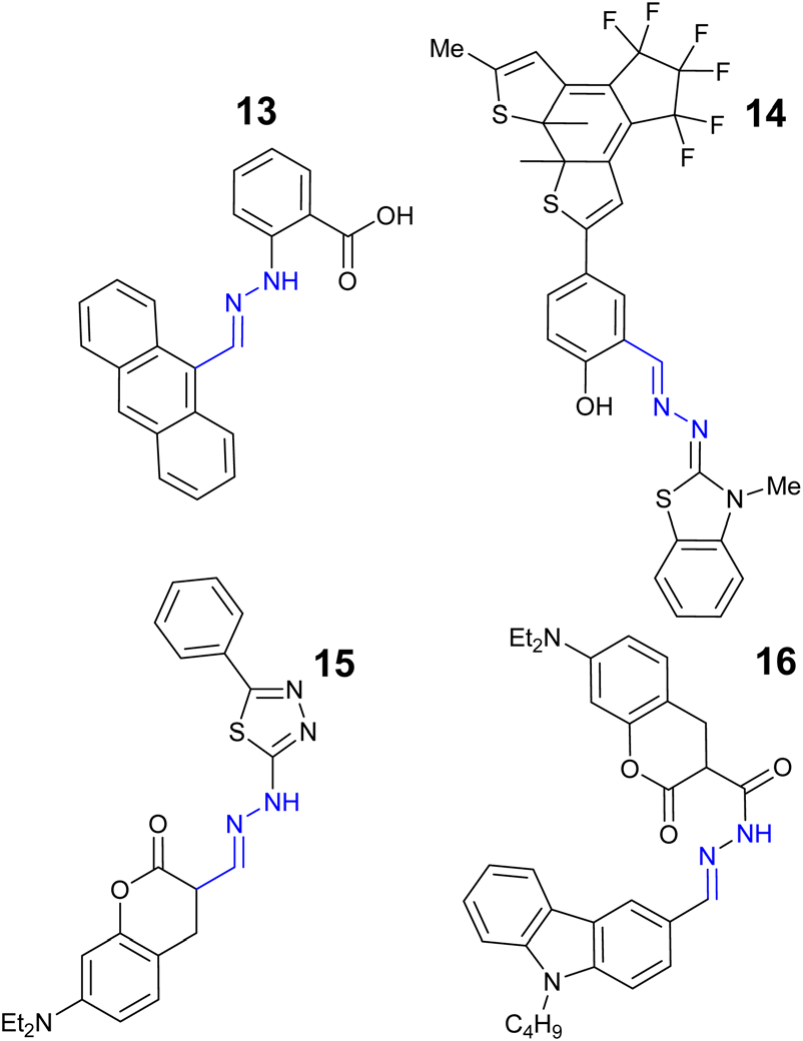
The structures of “turn on” Cu^2+^ sensors 13–16.^72–75^

Copper is the third most abundant transition metal in the human body^67^ and a key component of the immune system^68^ and central to the function of cytochrome C, a mitochondrial enzyme linked to cellular respiration.^69^ Excessive copper intakes can result in oxidative stress resulting in short term symptoms such as nausea and abdominal pain^70^ to long term conditions such as Parkinson's disease.^71^ The EU drinking water limit for copper is currently set at 31.7 μM^58^ and regular surveillance is essential. Anthracene derived sensor 13 (Fig. 10) displayed a strong 11-fold increase “turn on” response to Cu^2+^ at *λ*_em_ 455 nm due to ICT with LoD 0.53 nM in 2 : 1 DMSO : H_2_O solution.^72^ Real-world application of the detection of Cu^2+^ in sewage and tap water alongside the ability to extraction Cu^2+^ from the environment were reported.^72^ Benzothiazolinone–hydrazone 14 (Fig. 10) demonstrated a “turn off” signal with 1 : 1 binding to Cu^2+^ at *λ*_em_ 597 nm with LoD 84.0 nM.^73^ Quantification of Cu^2+^ in a range of river and tap water environments with >98% recoveries were reported.^73^ Thiadiazole based hydrazone 15 (Fig. 10) demonstrated high selectivity for Cu^2+^ with a “turn off” response at *λ*_em_ 540 nm, LoD 13.6 nM in 1 : 3 H_2_O : DMSO.^74^ Coumarin–hydrazone 16 (Fig. 10) was a “turn on” sensor for Cu^2+^ due to inhibition of PET with a 2 : 1 binding ratio with Cu^2+^ with LoD of 0.19 μM.^75^ Sensor 16 was shown to track and monitor Cu^2+^*in vitro* and detect Cu^2+^ in real-world river samples.^75^

**Fig. 11:**
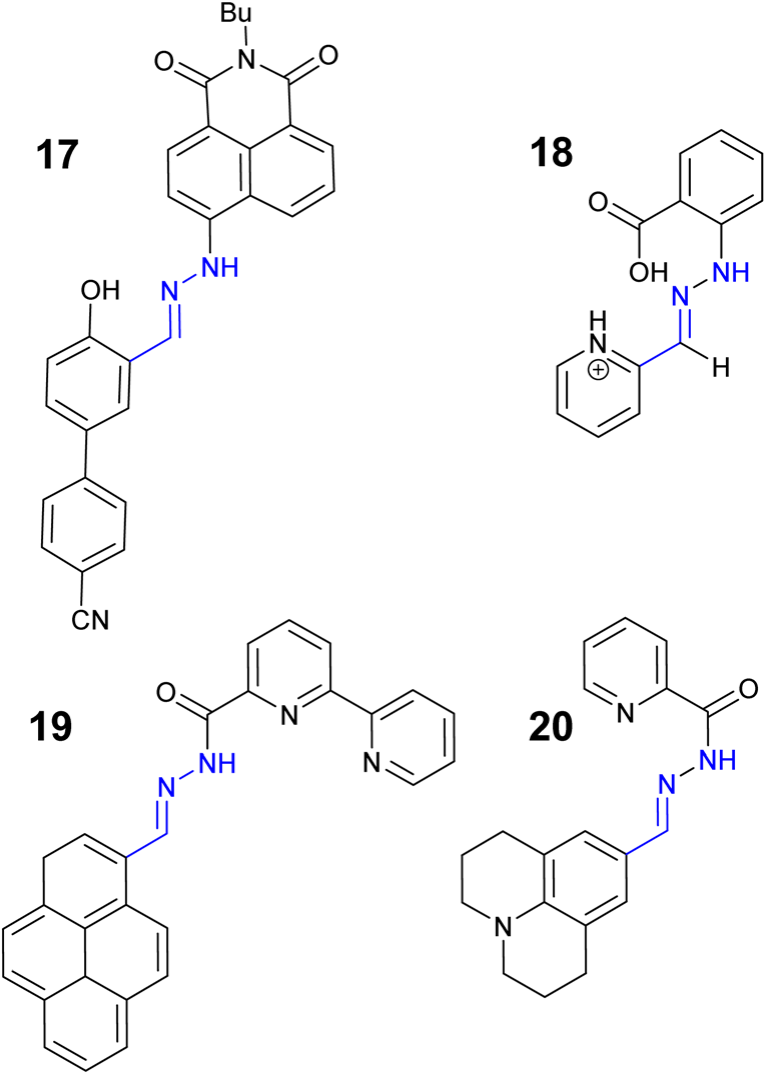
The structures of “turn on” Cu^2+^ sensors 17–20.^76–79^

1,8-naphthalimide–hydrazone 17 (Fig. 11) produced a “turn on” response to Cu^2+^ at *λ*_em_ 462 nm with 1 : 1 Cu^2+^ due to LCT with a LoD of 17 nM.^76^17 could detect Cu^2+^ in real-world samples such as beer and drinking water samples with high recoveries.^76^ Sensor 18 (Fig. 11) displayed a “turn off” response due to PET with a 1 : 1 ratio Cu^2+^ and a LoD of 0.9 μM in 4 : 1 H_2_O : MeCN.^77^ Pyrene–hydrazone 19 (Fig. 11) displayed a strong “turn on” signal with Cu^2+^ at *λ*_em_ 466 nm with a LoD of 0.66 μM in 8 : 2 H_2_O : MeCN solution.^78^ Sensor 19 displayed low cytotoxicity to Vero cells, a kidney cell line, confirming 19 can be used to track Cu^2+^ in living cells.^78^ Julolidine–hydrazone 20 (Fig. 11) was shown to give a “turn on” signal for Cu^2+^ in 1 : 1 DMSO : H_2_O solution at *λ*_em_ 420 nm with a LoD of 0.16 μM.^79^ Real-world validation for the detection of Cu^2+^ in canal, river and rainwater samples with excellent recoveries of >98% were reported for sensor 20.^79^

## 6 Sensors for Zn^2+^

**Fig. 12:**
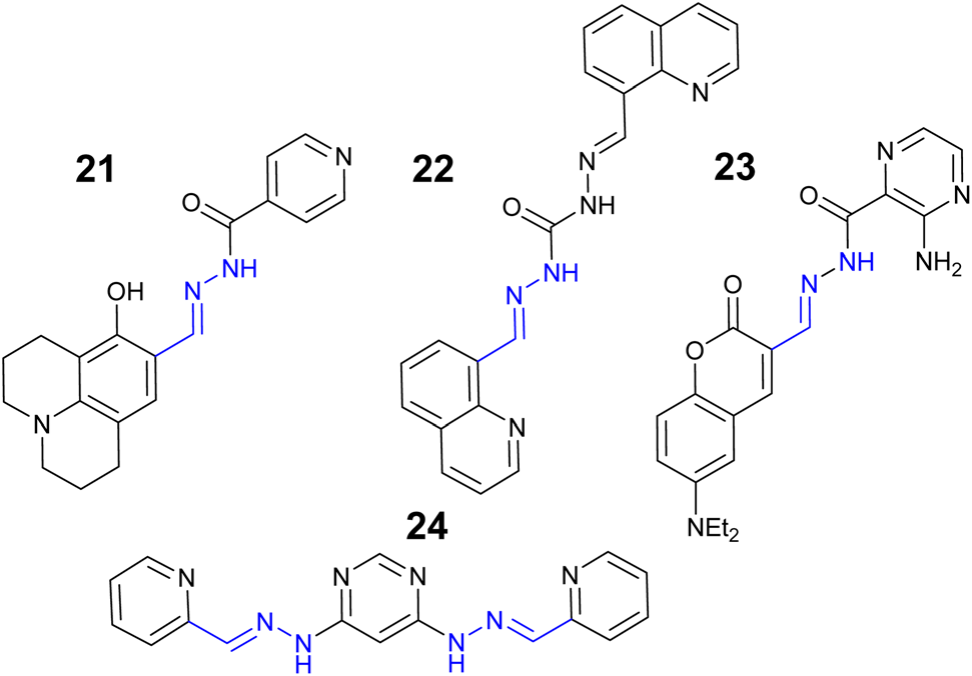
The structures of “turn on” Zn^2+^ sensors 21–24.^83–86^

Zinc is the second most abundant transition metal in the human body and critical to enzyme maintenance,^80^ gene expression and neurological functions.^81^ Excess and unregulated zinc is associated with Parkinsons and Alzheimer's disease with the WHO recommended drinking water limit set at 46 μM.^82^ The Julolidine–hydrazone, 21 (Fig. 12) is a “turn on” sensor for Zn^2+^ at *λ*_em_ 610 nm attributed to CHEF on 1 : 1 binding Zn^2+^ in 6 : 4 H_2_O : DMSO.^83^

Interestingly 21 did not response to Cu^2+^ despite its structural similarity to sensor 20 (Fig. 11) which was a “turn off” Cu^2+^ sensor. The potential of 21 as an INHIBIT logic gate and detection of Cu^2+^ in river and tap water real-world analysis confirmed.^83^ Quinoline–hydrazone 22 (Fig. 12) was a “turn on” Zn^2+^ sensor at *λ*_em_ 570 nm due to CHEF with a LoD of 0.66 μM in 4 : 6 H_2_O : MeOH solution.^84^ Confocal microscopy studies confirmed 22 can monitor Zn^2+^ in living systems.^84^ Coumarin based 23 (Fig. 12) was a “turn on” sensor for Zn^2+^ due to AIE with LoD 3.25 μM.^85^ The detection and monitoring of Zn^2+^*in vitro* in HeLa cells using confocal microscopy was confirmed for 23.^85^ Pyrimidine–hydrazone 24 (Fig. 12) bearing two pyridine units is a “turn on” sensor with a 2 : 1 Zn^2+^ to sensor ratio at *λ*_em_ 590 nm, LoD of 95.0 nM.^86^ This sensor also reported the ability to monitor Zn^2+^*in vitro* in the C2C12, a mouse myoblast, cell line.^86^ In summary hydrazone sensors are particularly attractive as “turn on” sensors for Zn^2+^.The large Stokes shift is advantageous for *in vitro* monitoring.

## 7 Sensors for Hg^2+^

**Fig. 13:**
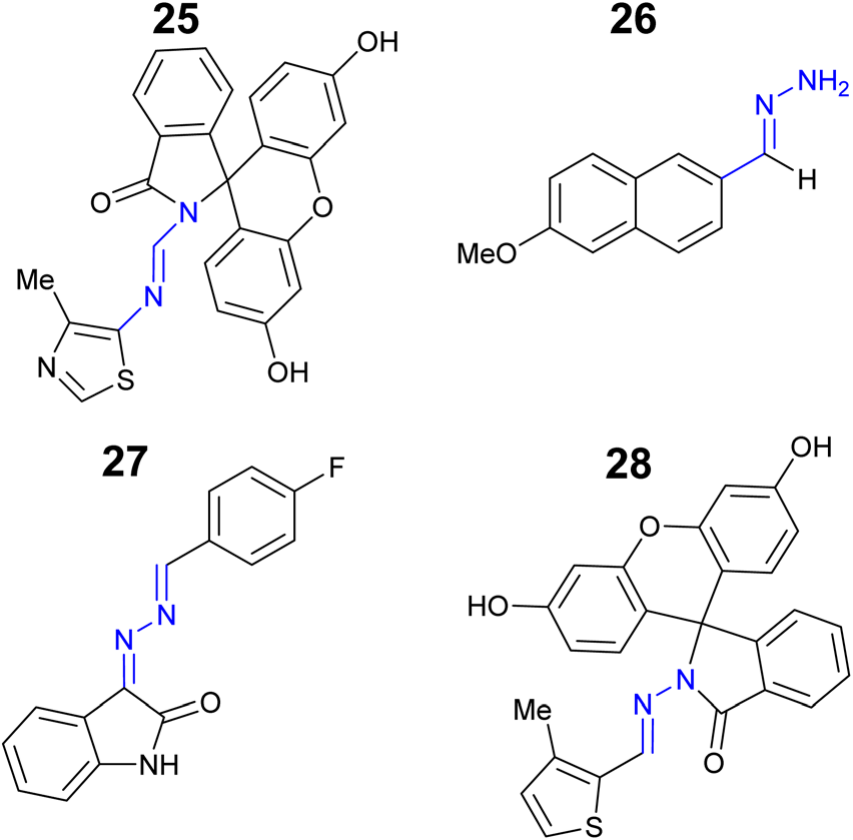
The structures of “turn on” Hg^2+^ sensors 25–28.^90–93^

Mercury is a rare element in the Earth's crust, often found as the Hg^2+^ ion in cinnabar (mercury sulfide).^87^ The neurotoxicity of mercury is well known resulting in one of the strictness exposure limits of all elements^88^ with the WHO maximum level in drinking water set at 5 nM.^44^ A particular challenge with mercury sensors is selective detection of Hg^2+^ over other group 12 metals for example Zn^2+^ and Cd^2+^. As a result, only a few Hg^2+^ specific sensors have been developed.^89^ Fluorescein based hydrazone 25 (Fig. 13) displayed an excellent Cd^2+^ specific “turn on” response at *λ*_em_ 520 nm with no observable change with Zn^2+^ or Cd^2+^.^90^ Sensor 25 had a LoD of 0.23 μM in 8 : 2 H_2_O : DMSO solution with the ability to monitor Hg^2+^*in vitro*.^90^ This sensor demonstrates it is possible to produce a useful Hg^2+^ sensor with real-world applications. Methoxynaphthalene–hydrazone 26 (Fig. 13) is a “turn on” Hg^2+^ sensor at *λ*_em_ 438 nm with a LoD of 6.0 μM in a 1 : 1 DMSO : H_2_O solution.^91^ Interestingly neither Zn^2+^ or Cd^2+^ interfered significantly with the “turn on” response to Hg^2+^ which was based on hydrolysis of the hydrazone.

Isatin derived hydrazone 27 is a “turn on” sensor for Hg^2+^ at *λ*_em_ 440 in 9 : 1 H_2_O : ethanol with a LoD of 3.6 μM and a 1 : 1 binding ratio.^92^ The fluorescein–hydrazone 28 was developed as a “turn on” Hg^2+^ sensor with a 1 : 1 binding ratio and LoD as low as 137 nM in a 1 : 9 H_2_O : solution.^93^ The application of 28 as an INHIBIT logic gate was confirmed.

## 8 Multi-analyte sensors

**Fig. 14:**
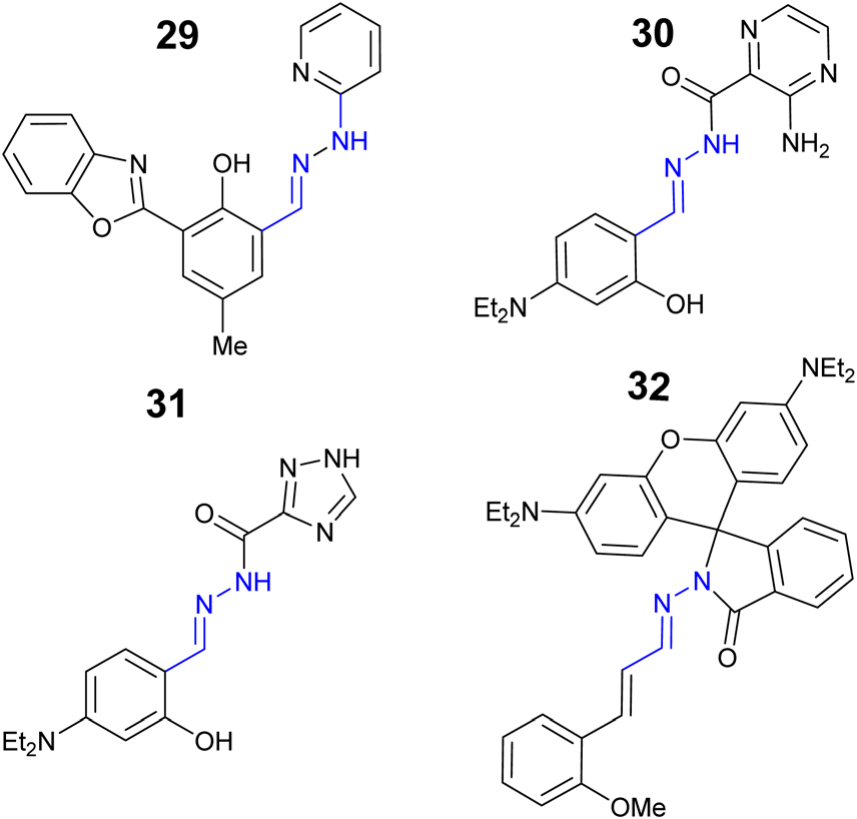
The structure of multi-analyte sensors 27–30.^94–97^

One rapidly emerging area of hydrazone-based sensors is the search for a single sensor which can detect multiple analytes, a multi-analyte sensor. Benzoxazole–hydrazone sensor 29 (Fig. 14) is capable of selectively detecting and distinguishing between three different trivalent analytes: Cr^3+^, Al^3+^ and Fe^3+^ in aqueous environments.^94^

Sensor 29 displayed a ESIPT “turn on” response to Cr^3+^ at *λ*_em_ 563 nm, “turn on” at *λ*_em_ 527 nm with Al^3+^ and “turn off” for Fe^3+^ at *λ*_em_ 620 nm. Benzoxazole–hydrazone 29 was able to monitor Cr^3+^*in vitro* in human mesenchymal stem cells demonstrating real-world application of this sensor.^94^ Pyrazine sensor 30 (Fig. 14) displayed multiple “turn on” functionality for Zn^2+^ at *λ*_em_ 545 nm, “turn on” for Al^3+^ at *λ*_em_ 525 nm and Mg^2+^ at *λ*_em_ 600 nm due to inhibition of ESIPT.^93^ The monitoring of Al^3+^ in HeLa cells was confirmed using confocal microscopy.^95^ Triazole–hydrazone 31 (Fig. 14) is a “turn on” sensor for Al^3+^, LoD 22.5 nM and Zn^2+^ at *λ*_em_ 460 due to CHEF, LoD 102.5 nM.^96^ Sensor 31 was confirmed to detect and monitor Zn^2+^ in HeLa cells *via* confocal microscopy.^96^ Rhodamine sensor 32 (Fig. 14) was developed as a dual “turn on” sensor for Al^3+^ at *λ*_em_ 588 nm and Cu^2+^ at *λ*_em_ 580 nm with LoD of 8.3 nM and 0.29 μM respectively.^97^

## 9 Conclusions

2014–2024 has been a fruitful decade for the development of hydrazone based fluorescent sensors for the detection of multiple toxic metals in human health. The hydrazone functional group enables the combination of well-established fluorophore and chelation units into a single sensor for improved properties. Typical examples include the hybridisation of established fluorescent dyes such as rhodamine and fluorescein with nitrogen-based chelators such as pyridine and pyrazine. The ease of hydrazone synthesis, typically a single step, from commercially available carbonyl and hydrazine starting materials easily facilitates this fusion approach to sensor development. Hydrazone sensors operate through a multitude of fluorescence pathways including AIE, PET, CHEF, CHEQ, ESIPT and FRET. Of the hydrazone sensors reviewed over the past decade, the vast majority are aqueous soluble ensuring these sensors find real-world applications. The detection of toxic metals in the environment, for example river and drinking water samples and *in vitro* monitoring of metals in cell cultures provide firm validation of the hydrazone scaffold. Current limit of detections within or below legal limits confirming they have a useful role to play in heavy metal surveillance. Recent advances in the development of multi-analyte sensors are likely to accelerate allowing researchers to detect and monitor multiple analytes concurrently. There are major challenges ahead, for example the develop of Hg^2+^ specific sensors with LoD below the drinking water limit of 5 nM and the detection of the group 1 and 2 biological metals *in vitro*. Nevertheless, hydrazone sensors will continue to be a vital addition to the sensing toolkit for the foreseeable future.

## Data availability

No primary research results, software or code have been included and no new data were generated or analysed as part of this review.

## Conflicts of interest

There are no conflicts to declare.
